# *In vivo* Cigarette Smoke Exposure Decreases CCL20, SLPI, and BD-1 Secretion by Human Primary Nasal Epithelial Cells

**DOI:** 10.3389/fpsyt.2015.00185

**Published:** 2016-01-13

**Authors:** James Jukosky, Benoit J. Gosselin, Leah Foley, Tenzin Dechen, Steven Fiering, Mardi A. Crane-Godreau

**Affiliations:** ^1^Department of Natural Science, Colby-Sawyer College, New London, NH, USA; ^2^Department of Otolaryngology, Dartmouth Hitchcock Medical Center, Lebanon, NH, USA; ^3^Department of Microbiology and Immunology, Geisel School of Medicine at Dartmouth, Lebanon, NH, USA

**Keywords:** cigarette smoke exposure, primary nasal epithelium, antimicrobial peptides, CCL20, beta defensing-1, SLPI, innate immune response, nasal epithelial cell culture

## Abstract

Smokers and individuals exposed to second-hand cigarette smoke have a higher risk of developing chronic sinus and bronchial infections. This suggests that cigarette smoke (CS) has adverse effects on immune defenses against pathogens. Epithelial cells are important in airway innate immunity and are the first line of defense against infection. Airway epithelial cells not only form a physical barrier but also respond to the presence of microbes by secreting antimicrobials, cytokines, and chemokines. These molecules can lyse infectious microorganisms and/or provide signals critical to the initiation of adaptive immune responses. We examined the effects of CS on antimicrobial secretions of primary human nasal epithelial cells (PHNECs). Compared to non-CS-exposed individuals, PHNEC from *in vivo* CS-exposed individuals secreted less chemokine ligand (C-C motif) 20 (CCL20), Beta-defensin 1 (BD-1), and SLPI apically, less BD-1 and SLPI basolaterally, and more CCL20 basolaterally. Cigarette smoke extract (CSE) exposure *in vitro* decreased the apical secretion of CCL20 and beta-defensin 1 by PHNEC from non-CS-exposed individuals. Exposing PHNEC from non-CS exposed to CSE also significantly decreased the levels of many mRNA transcripts that are involved in immune signaling. Our results show that *in vivo* or *in vitro* exposure to CS alters the secretion of key antimicrobial peptides from PHNEC, but that *in vivo* CS exposure is a much more important modifier of antimicrobial peptide secretion. Based on the gene expression data, it appears that CSE disrupts multiple immune signaling pathways in PHNEC. Our results provide mechanistic insight into how CS exposure alters the innate immune response and increases an individual’s susceptibility to pathogen infection.

## Introduction

There is copious evidence that exposure to primary cigarette smoke (CL) and/or second-hand cigarette smoke (SHCS) is associated with increased frequency of infections or other symptoms of perturbations of the immune system. Exposure to primary CL or SHCS is a risk factor for recurrent otitis media, upper respiratory tract infection (1, 2), meningococcal infection (3), bacteria pneumonia, oral candidiasis (4, 5), type 2 herpes simplex virus (6), pneumococcal disease, influenza (7), asthma (8–11), and rhinosinusitis (12). One recent review of the epidemiology of smoke exposure and infection concludes that “Cigarette smoking is a substantial risk factor for important bacterial and viral infections” (7).

Cigarette smoke exposure alters the physiology of the upper airway. Evaluation of adenoid tissue removed from children living in SHCS-contaminated environments demonstrates significant histopathological and ultrastructural differences in these upper airway immune tissues when compared to children not exposed to SHCS (1). In studies of the effect of CS-extract on adherence of respiratory pathogens to buccal epithelial cells, CS-extract increased bacterial binding to the cells (13). Upper airways of CS-exposed individuals harbor more potential pathogens than those of non-smokers (14). These findings are consistent with studies in mice. For example, when mice exposed to CS were compared to mice with sham exposure, CS-exposed mice were less able to clear *Pseudomonas aeruginosa*. Besides the delayed rate of clearance, mice exposed to CS experienced increased inflammation (15). In studies looking at changes in immune responses to bacterial challenge in mice exposed to nicotine, exposed mice exhibited significantly higher titers of influenza virus following infection (16). Subsequent studies of human monocytes exposed to bacterial toxins and then to cigarette smoke extract (CSE) demonstrated extremely aberrant immune responses relative to cells exposed to bacterial toxins but not exposed to CSE (17).

A major function of the mucosal epithelium is innate immune protection (18, 19). Beyond providing a physical barrier between the external environment and the body itself in the digestive, reproductive, and respiratory organs, the epithelium produces a complex multifaceted mucus layer that lines mucosal surfaces (20). To maintain balance with the body’s normal flora and to suppress infections, epithelial cells monitor their environment and respond to microbial challenges by altering secretions into the luminal environment. Partners with the epithelial cells in the delicate balancing act with microbial flora are leukocytes that can also recognize a microbial threat and respond by altering secretions. In response to threatening microbes recognized by epithelial cells and/or by leukocytes, airway mucosal epithelium produce antimicrobial proteins, including defensins, lysozyme (LYZ), lactoferrin (LF), secretory leukocyte protease inhibitor (SLPI), and chemokine ligand (C–C motif) 20 (CCL20) (19, 21–23). LYZ is a cationic bacteriolytic protein produced by mucosal epithelial cells, another cationic protein is LF that impedes bacterial growth and replication by sequestering iron. LYZ and LF are both released by the nasal epithelium (24). SLPI, a low molecular weight protease inhibitor with an antimicrobial domain is also found in nasal secretions (25, 26). Small cationic peptides, including defensins and defensin-like CCL20, exert their effect through electrostatic interactions with bacterial membranes that puncture the microbial cell. Recently, CCL20 has also been found to exhibit antiviral activity as well (27). In addition to antimicrobial activity, many of these peptides, including human beta-defensin 2 (BD2) and CCL20, also serve as chemokines to recruit specialized immune cells carrying the CCR6 receptor to the site of an infection (24, 28, 29).

The airway mucosa produces antimicrobials both constitutively and in response to recognition of microbes. To recognize potential pathogens, the innate immune system relies on *pathogen associated molecular patterns* (PAMP) that are unique to microbes. One group of receptors that recognize PAMP is the toll-like receptors (TLR) that are expressed on epithelial cells as well as most specialized immune cells. PAMP ligands are repeated in a wide variety of pathogenic microbes, including bacteria, fungi, and viruses. TLR ligands include lipoteichoic acid (LTA), derived from Gram-positive bacteria (30). Common to TLR in mammals is the ability to induce effects through activation of the transcription factor NFκB, and through mitogen-activated protein kinases (MAPKs) independent of NFκB (31). TLR activation results in an induction of many molecules, including antimicrobials and cytokines necessary for innate and adaptive immune protection (16, 32, 33).

As noted above, it is clear that CS perturbs the response to pathogens. Although various experiments have associated CS exposure with specific immune system perturbations, the mechanisms involved are not yet fully understood. An understanding of those mechanisms will provide new prognostic, diagnostic, and therapeutic approaches to smoke-related morbidity and mortality. The experiments reported here tested the hypothesis that CS exposure *in vivo* or *in vitro* suppresses the release of antimicrobial peptides from primary human epithelial cells.

Our group has shown that *in vitro* CSE exposure reduces production of the antimicrobial peptide CCL20 in Beas-2b, immortalized human bronchial epithelial cells (34). However, Beas-2b, although not transformed, is a line that has been maintained in culture for long periods and is a genetic representation of a single individual. In order to examine the effect of CS exposure on antimicrobial peptide secretion in true primary cells, we established short-term cultures of primary human nasal epithelial cells (PHNECs) from smoke-naïve individuals and smokers, and assayed antimicrobial peptide secretion.

## Materials and Methods

### Influence of *In vivo* Cigarette Smoke Exposure on the Antimicrobial Secretions of PHNEC

Beta-defensin 1 (BD1), CCL20, and SLPI secretion were assayed in cells obtained from CS-exposed individuals and individuals with no CS exposure. PHNECs were obtained from 13 individuals with no CS exposure and 7 individuals with significant primary CS or SHCS exposure. PHNECs were obtained and cultured as described in section below. As noted by other groups studying PHNEC, it was much more difficult to culture PHNEC from CS-exposed individuals compared to non-CS-exposed individuals (Carson, personal communication). Four experimental treatments were performed using both *in vivo* smoke-exposed PHNEC and cells from smoke-naïve individuals. These treatments were constitutive secretion where nothing was applied, CSE exposed, LTA stimulation (LTA stimulated) to simulate a bacterial infection, and CSE exposed with LTA stimulation. CSE-exposed primary human nasal cell cultures were treated apically with 300 μL 1× CSE in air–liquid interface (ALI) for 3 h, other treatments received ALI only. This CSE exposure did not increase cell death significantly 24 h later (data not shown). CSE was then removed by suction and cells were rinsed twice with PBS. Then LTA-exposed cell cultures were stimulated apically with LTA from *Bacillus subtilis* (10 μg/mL) in ALI media by applying 300 uL in the apical compartment. PHNECs were incubated for 20 h, and after this period apical and basolateral supernatants were harvested. Cell supernatants were centrifuged at 17,000 × *g* for 10 min and the supernatants were removed and stored at −80°C. BD1, secretory leukocyte protease inhibitor (SLPI), and CCL20 were assayed by ELISA in apical and basolateral secretions either using commercially prepared assay kits or ELISAs developed from ELISA development kits (R&D systems, Mckinley, MN, USA, or Leinco Technologies, St. Louis, MO, USA).

A two-way analysis of variance (ANOVA) was used to examine the effect of treatment (constitutive, CSE exposed, LTA stimulated, and CSE exposed with LTA stimulation) or smoking status (*in vivo* smoke-exposed versus cells from smoke-naïve individuals) and any interactions between treatment and smoking status on PHNEC secretions. Data were natural log (ln) transformed for analysis to meet the underlying ANOVA assumption of a normal distribution. *Post hoc* analyses were carried out with Tukey’s HSD tests. All statistics and data transformations were performed using JMP 11.

### PHNEC Culture

Primary human nasal epithelial cells were obtained from smoke-exposed and non-smoke-exposed healthy human volunteers and differentiated *in vitro* in transwells as described by Carson et al. (35). The criteria for recruiting subjects were similar to those described previously (35, 36). We determined smoke exposure status via questionnaire and confirmed it through measurement of urine cotinine.

Primary human nasal epithelial cells were collected from healthy smoking and non-smoking adult volunteers by gently scraping the inferior surface of the turbinate five to eight times with a Rhinopro™ curette (Arlington Scientific, Arlington, TX, USA). The curette was inserted through a nasoscope, which was used to visualize the inferior turbinate. This protocol was approved by the Institutional Review Board of the Geisel School of Medicine at Dartmouth.

Primary human nasal epithelial cells from nasal scrapes were seeded on human collagen-coated wells of a 12-well plate and grown to 70% confluence in ALI media (Lonza Biologics). At 70% confluence, PHNECs were trypsinized and seeded into flasks and grown in media that was one part ALI and two parts bronchial epithelial growth medium (BEGM) (Lonza Biologics). PHNEC in flask were grown to 70% confluency and subsequently trypsinized and seeded into collagen-coated filter supports with a 0.4-μM pore size (Trans-CLR; Costar, Cambridge, MA, USA) and grown in ALI media. We promoted mucociliary differentiation of PHNEC after cells grew to confluency by supplementing the media with all-trans retinoic acid and the media was removed from the apical compartment to create ALI culture conditions. We observed mucociliary differentiation 14–21 days after ALI differentiation and the cultures of PHNEC were utilized experimentally at this stage.

### Smoke Extract Generation

Cigarette smoke extract was generated by using vacuum suction to draw the smoke from a single research reference grade cigarette (Kentucky Cigarette Research and Development Center at the University of Kentucky, Lexington, KY, USA) through 100 mLs of ALI media in a controlled manner.

### Time-Course Experiments

Primary human nasal epithelial cells were obtained from four non-smoke-exposed individuals, expanded, and differentiated as described previously. Constitutive, CSE, LTA, and LTA + CSE treatments were established as described previously. Three wells of cells per individual were used in each treatment. At time points of 1 and 6 h after LTA stimulation, we harvested cells apical and basolateral supernatants and RNA (for time-course gene expression). Apical and basolateral BD1 and CCL20 secretion were measured using ELISA. A repeated measures MANOVA was used to test for differences in CCL20 and BD-1 secretion between the four treatments previously described and time points (1 and 6 h post-LTA stimulation). Bonferroni-corrected *t*-tests were used *post hoc* to identify significant differences between specific treatments.

Expression of 511 immunology-related genes was quantified using Nanostring technology. We analyzed expression at the 1 and 6 h time point post-LTA stimulation and compared LTA and CSE + LTA treatments. RNA was extracted from individual transwells (*n* = 12) using a Qiagen Allprep kit. The total counts of mRNAs were assayed using nCounter GX Human Immunology gene expression code set that uses molecular barcodes attached to target-specific probes. Barcoded probes hybridize directly to their RNA targets in solution, and the probes are counted directly using microscopic imaging. Following the manufacturer’s protocol, 100 ng mRNA was hybridized with the nCounter GX Human Immunology code set and loaded into the nCounter prep station followed by imaging and quantification using the nCounter Digital Analyzer. Quality control and data normalization were performed using the nSolver analysis software. Data were normalized to global means for internal positive controls, and subsequently normalized to the geometric means of a suite of housekeeping genes. Two reference genes (GUSB and HPRT1) were eliminated from the normalization panel because their average raw count data varied more than 1 SD between LTA and CSE + LTA treatments. The panel of genes used for normalization included genes with both low and high expression levels.

Specific samples from each individual were paired when experiments were designed and assigned to separate treatments. Gene expression in the LTA-stimulated treatment and the LTA + CSE treatments were compared by matched pair *t*-test separately at each time point. No corrections were made for multiple comparisons as each barcode is considered an independent assay.

## Results

### PHNECs from CS-Exposed Individuals Were Difficult to Culture

**Figure 1:**
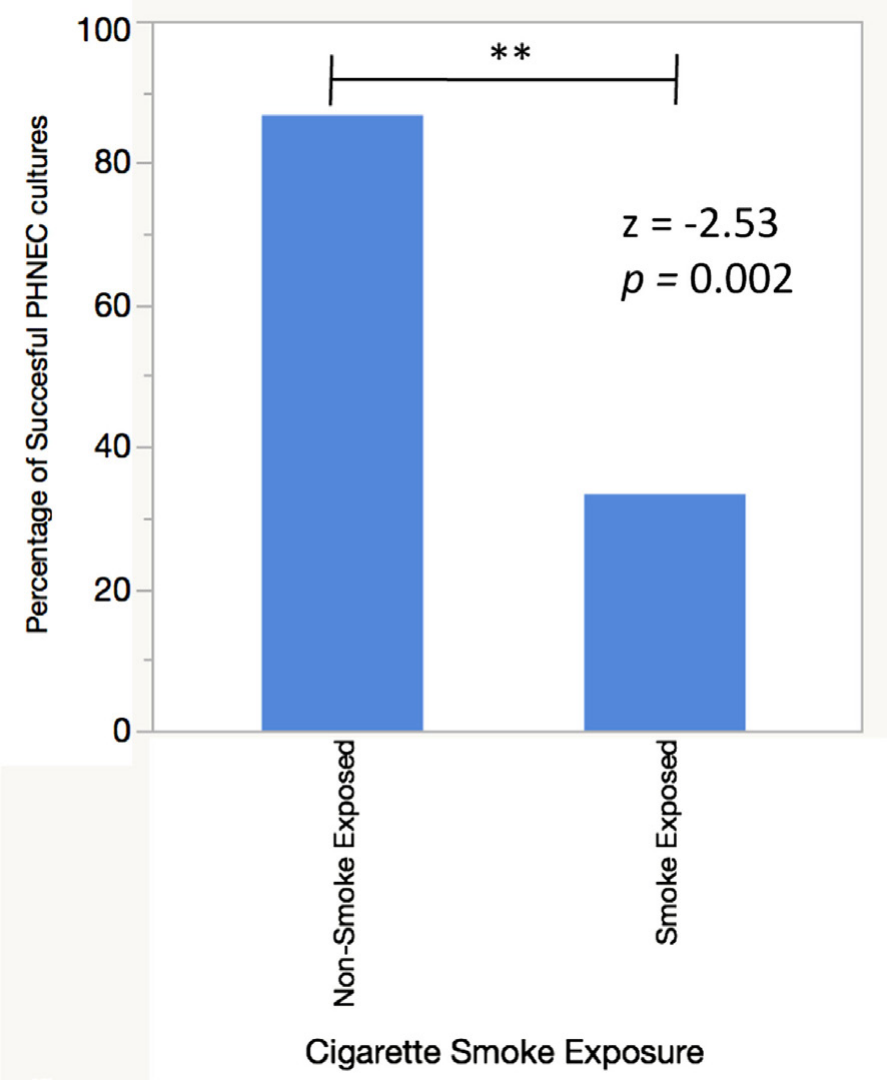
**PHNEC sampled from cigarette smoke-exposed individuals fail to grow into confluent ALI cultures more often than PHNEC from non-smoke-exposed individuals**. A successful culture was defined as one that grew to confluency in a transwell and differentiated into a ciliated, mucus-producing air–liquid interface culture.

To examine the difference in antimicrobial secretions between PHNEC from smokers and non-smokers and their response to CSE exposure, cultures were established from each group. While PHNEC from non-smokers grew readily, PHNEC from smokers generally grew poorly and only a fraction of the donors provided cells that could be grown to the required level for the assays. Here, 86.6% of nasal scrapes from healthy donors (*n* = 15) were expanded in culture and differentiated into ciliated ALI cultures as described in our methods, while 33.3% of nasal scrapes from CS and SHS exposed individuals (*n* = 12) were able to grow and differentiate in the same manner. There was a significant difference between successful non-CS-exposed and CS-exposed PHNEC cultures using a *z*-test to compare two population proportions (*z* = −2.83, *p* = 0.002, Figure 1). These results reflect outcomes from a subset of nasal samples that were gathered after noticing this trend. This is similar to what has been reported by other researchers harvesting PHNEC from smoke-exposed individuals (Carson, personal communication).

### *In vivo* Smoke Exposures Alters Antimicrobial Peptide Secretions by PHNEC

#### Chemokine Ligand (C–C Motif) 20

**Figure 2:**
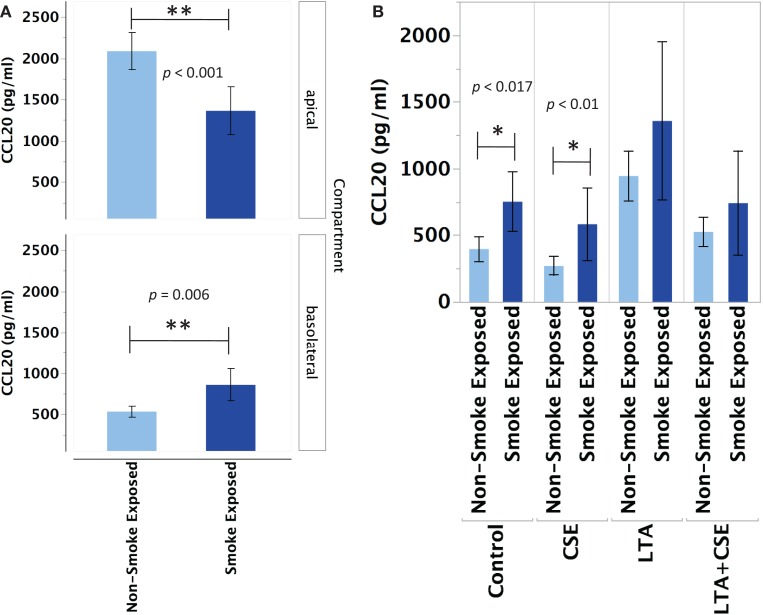
**CCL20 secretion is altered in PHNEC from *in vivo* smoke-exposed individuals**. Compared to non-smoke-exposed individuals, smoke-exposed individuals had significantly lower apical CCL20 secretion and significantly higher basolateral CCL20 secretion **(A)**. Figure 2A shows secretion data pooled (from all treatments) from every PHNEC secretion sample. CCL20 secretion into the basolateral compartment is significantly higher in PHNEC from *in vivo* smoke-exposed individuals in the control (constitutive) and the CSE-exposed treatments **(B)**.

Primary human nasal epithelial cell from *in vivo* CS-exposed individuals secreted significantly less CCL20 apically and significantly more CCL20 basolaterally than PHNEC from non-CS-exposed individuals. Figure 2A illustrates this finding by pooling all treatment groups and compares PHNEC CCL20 secretion from non-CS and *in vivo* smoke-exposed individuals. Our measurements of CCL20 in the apical compartment showed that there was no significant effect of treatment (constitutive, CSE exposed, LTA stimulated, and CSE exposed with LTA stimulation) and no interaction between treatment and smoking status. However, there was a significant effect of smoking status on CCL20 secretion and this was highly significant in both the apical (*F*_1,235_ = 24.07, *p* < 0.0001) and basolateral (*F*_1,171_ = 12.2, *p* < 0.001) compartments. Regardless of the treatment group, *in vivo* smoke-exposed PHNEC secreted less CCL20 apically and more CCL20 basolaterally than non-smoke-exposed individuals (Figure 2A). There was a significant effect of treatment in the basolateral compartment (*F*_3,171_ = 3.19, *p* = 0.025). *Post hoc* analysis showed significantly increased CCL20 secretion in *in vivo* smoke-exposed PHNEC in the basolateral constitutive (*p* = 0.017) and basolateral CSE (*p* = 0.01) treatments (Figure 2B).

#### SLPI

**Figure 3:**
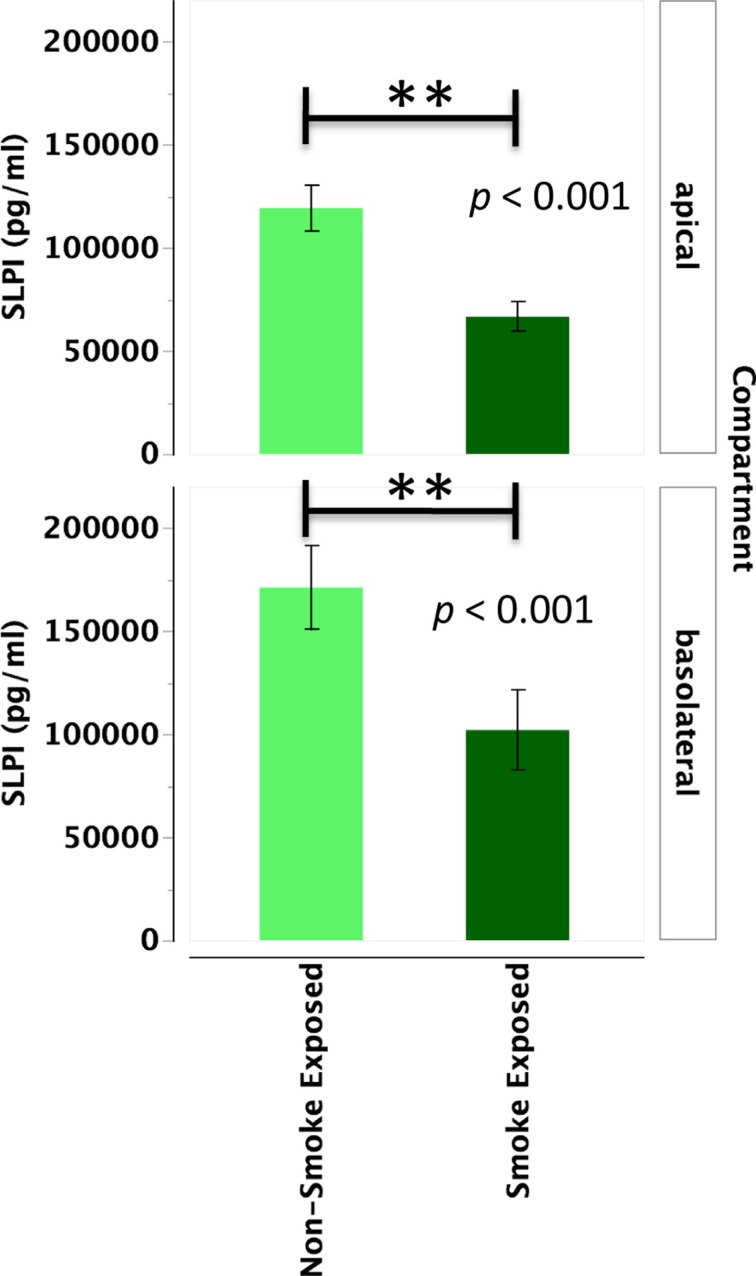
**SLPI secretion into the apical and basolateral compartments is significantly lower in PHNEC from *in vivo* smoke-exposed individuals**. SLPI secretion was not significantly different among the four treatments in our PHNEC experiments. However, there was a significant effect of smoke exposure status and this figure shows secretion data pooled (from all treatments) from every PHNEC secretion sample.

Primary human nasal epithelial cell from *in vivo* CS-exposed individuals secreted significantly less SLPI apically and basolaterally than PHNEC from non-CS-exposed individuals. Figure 3 illustrates this finding by pooling all treatment groups and compares PHNEC SLPI secretion from non-CS and *in vivo* smoke-exposed individuals. For SLPI from cultured PHNEC, there was a highly significant effect of smoking status on SLPI secretion but no significant effect of treatment and no interaction between treatment and smoking status. PHNEC from non-smoke-exposed individuals had much higher SLPI secretions in both the apical (*F*_1,139_ = 15.2, *p* < 0.001) and basolateral (*F*_1,154_ = 25.9, *p* < 0.001) compartments (Figure 3). Regardless of the treatment group, PHNEC from smoke-exposed individuals secreted less SLPI apically and basolaterally than that from non-smoke-exposed individuals.

#### Beta-Defensin 1

**Figure 4:**
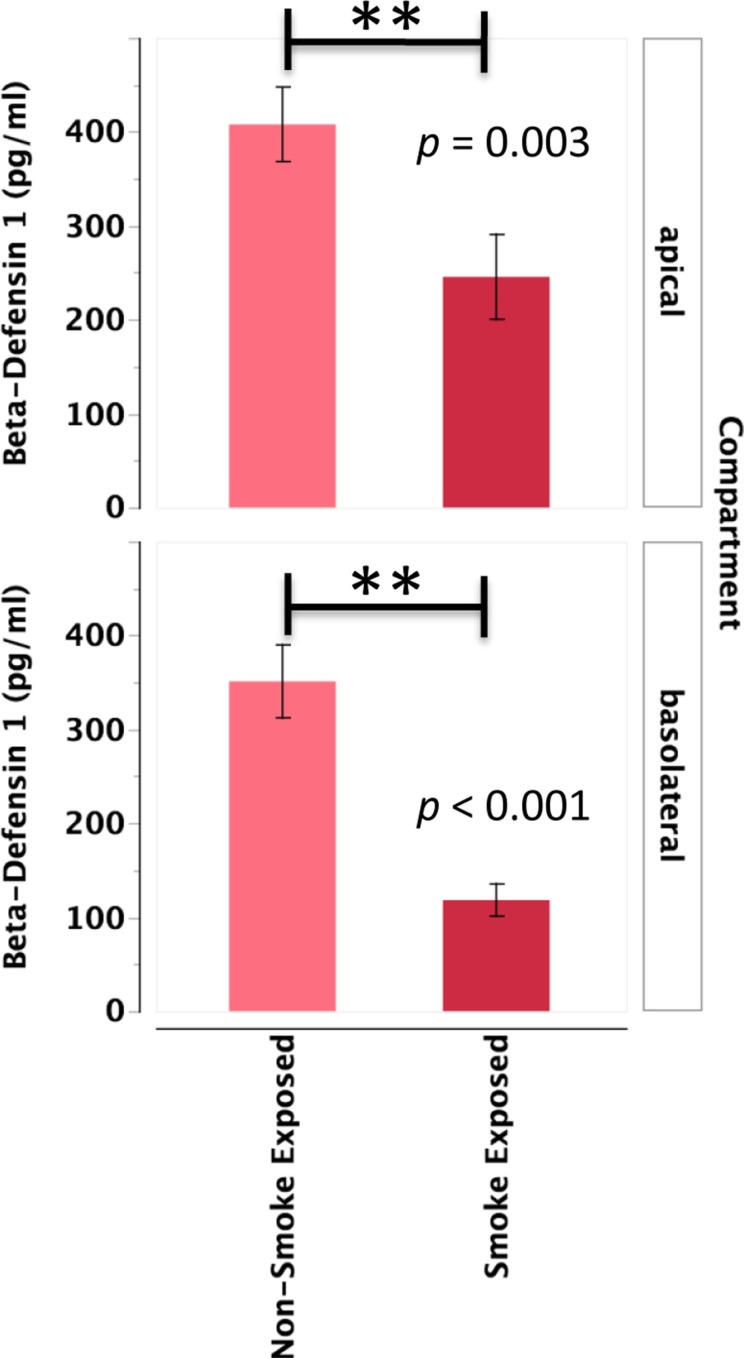
**Beta-defensin 1 secretion into the apical and basolateral compartments is significantly lower in PHNEC from *in vivo* smoke-exposed individuals**. Beta-defensin 1 secretion was not significantly different among the four treatments in our PHNEC experiments. However, there was a significant effect of smoke exposure status and this figure shows secretion data pooled (from all treatments) from every PHNEC secretion sample.

Primary human nasal epithelial cell from *in vivo* CS-exposed individuals secreted significantly less BD-1 apically and basolaterally than PHNEC from non-CS-exposed individuals. Figure 4 illustrates this finding by pooling all treatment groups and compares PHNEC BD-1 secretion from non-CS and *in vivo* smoke-exposed individuals. However, identical to SLPI, analysis of apical and basolateral BD-1 secretion also showed no significant effect of treatment and no interaction between treatment and *in vivo* smoking status. PHNEC cultured from *in vivo* smoke-exposed individuals secreted significantly less BD-1 into the apical (*F*_1,155_ = 8.92, *p* = 0.003) and basolateral compartments (*F*_1,167_ = 23.1, *p* < 0.001) (Figure 4).

### Time-Course Experiments

#### CSE Decreased Apical CCL20 Secretion in Unstimulated PHNEC at Hour 1 and LTA-Stimulated Cells at Hour 6 and Also Decreased Basolateral Secretion

**Figure 5:**
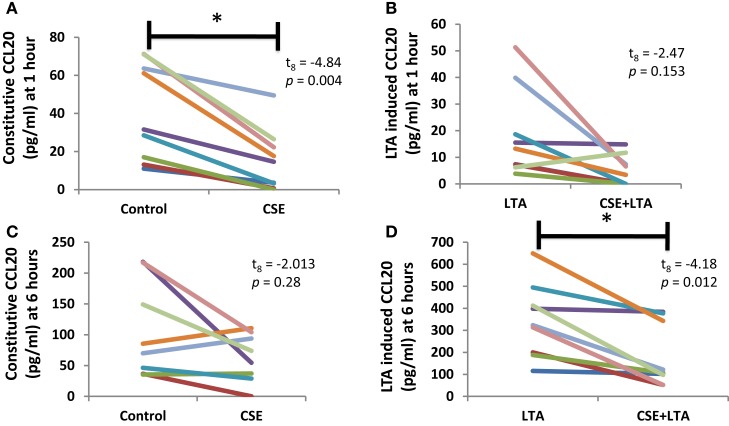
**CSE exposure significantly decreased constitutive apical CCL20 secretion at hour 1 (A) and LTA-stimulated apical CCL20 secretion at hour 6 (B)**. Apical CCL20 secretion from PHNECs from non-smoke-exposed individuals are shown at 1 h **(A,B)** and 6 h **(C,D)** post-LTA stimulation. Primary human nasal cell cultures were exposed apically to cigarette smoke extract in ALI media for 3 h, LTA and control treatments received ALI media only. CSE was then removed by suction and cells were rinsed twice with PBS. Subsequently, LTA and CSE + LTA treatments were stimulated apically with LTA from *B. subtillis* (10 μg/mL) in ALI media by applying 300 uL in the apical compartment. Supernatants were harvested at 1 and 6 h post-LTA stimulation. CCL20 was quantified by ELISA.

**Figure 6:**
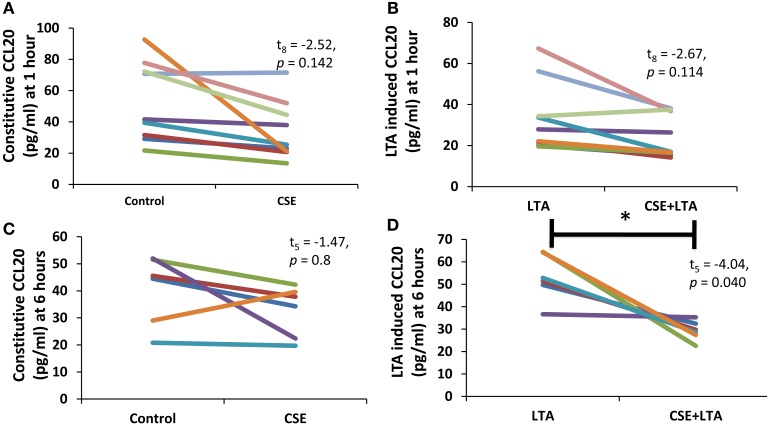
**PHNEC basolateral secretion of CCL20 significantly decreased following CSE exposure at the 6-h time point in LTA-induced samples**. Depicted in this figure are basolateral CCL20 secretions from PHNECs cultured from non-smoke-exposed individuals at 1 **(A,B)** and 6 h **(C,D)** post-LTA stimulation. PHNECs were exposed apically to cigarette smoke extract in ALI media for 3 h, LTA and control treatments received ALI media only. CSE was then removed by suction and cells were rinsed twice with PBS. Subsequently, LTA and CSE + LTA treatments were stimulated apically with LTA from *B. subtillis* (10 μg/mL) in ALI media by applying 300 uL in the apical compartment. Supernatants were harvested at 1 and 6 h post-LTA stimulation. CCL20 was quantified by ELISA.

Cigarette smoke extract exposure significantly decreased constitutive apical CCL20 secretion at hour 1 (Figure 5A), but this difference was not significant at hour 6 (Figure 5B). CSE exposure significantly decreased LTA-stimulated apical CCL20 secretion at hour 6 (Figure 5D), but this difference was not significant 1 hour post-LTA stimulation (Figure 5C). Basolateral secretion of CCL20 was significantly reduced by CSE exposure at the 6 hour time point in LTA-induced samples (Figure 6D), but not at 1-h post-LTA stimulation (Figure 6C). Constitutive basolateral secretion of CCL20 was not significantly altered by CSE at the 1-h and 6-h time points (Figures 6A,B).

Apical CCL20 secretion was measured in each treatment at 1 and 6 h time points; we observed a significant effect of time point (between subjects, *F*_1,16_ = 28.4, *p* < 0.001), treatment (within subjects, *F*_3,14_ = 16.27, *p* < 0.001), and interaction between time points (within subjects, *F*_3,14_ = 11.15, *p* < 0.001), and treatment for apical CCL20 secretion. Basolateral secretion of CCL20 showed no significant effect of time point but there was a significant effect of treatment (within subjects, *F*_3,11_ = 12.77, *p* < 0.001) and significant interaction between time point and treatment (within subjects, *F*_3,11_ = 6.13, *p* = 0.011).

#### No Differences Were Detected in BD-1 Secretion at the 1- or 6-h Time Points

No significant differences in apical or basolateral secretion of BD-1 were detected between CSE treated and untreated samples at the 1- or 6-h time point. We observed a significant effect of treatment with apical (within subjects, *F*_3,17_ = 5.043, *p* = 0.011), and basolateral (within subjects, *F*_3,17_ = 7.78, *p* = 0.004) BD-1 secretion. However, in *post hoc* analysis with Bonferroni corrections, no comparisons between CSE treated and untreated samples were significantly different at either time point (data not shown).

#### CSE Exposure Alters the Expression of Immune Related Genes after 3 h of CSE Exposure Followed by 1 h of LTA Stimulation

In order to further explore gene expression changes from CS exposure a set of 511 immunology-related genes were assayed by Nanostring technology. Total amounts of 20 immunology-related gene transcripts were significantly altered by CSE exposure out of 511 genes assays. This difference was measured in the first hour after LTA stimulation. Three genes had increased number of transcripts and 17 were significantly decreased. By 6 h after LTA stimulation, these differences were minimal and a significant increase in the transcripts of only one other gene was detected (Table 1).

**Table 1:** **Changes in gene expression in comparisons of cigarette smoke extract and unexposed treatments of LTA-stimulated PHNEC from healthy individuals**.

Time point	Gene name	Mean difference with CSE exposure	Change in expression with CSE exposure	*t* ratio	Degrees of freedom	*p*-value
Hour 1	ATG16L1	−26.18	Decrease	−2.83831	8	0.0219
Hour 1	CD59	−652.93	Decrease	−2.87	8	0.0208
Hour 1	CDKN1A	−919.37	Decrease	−2.31452	8	0.0493
Hour 1	CEACAM1	10.83	Increase	3.379778	8	0.0096
Hour 1	GPI	41.24	Increase	3.13942	8	0.0138
Hour 1	IFIH1	−25.28	Decrease	−2.71971	8	0.0263
Hour 1	GUSB	−17.83	Decrease	−4.01185	8	0.0039
Hour 1	IFNAR1	−2.96	Decrease	−2.96357	8	0.0180
Hour 1	IGF2R	−29.66	Decrease	−3.98707	8	0.0040
Hour 1	IRAK1	−49.62	Decrease	−3.57147	8	0.0073
Hour 1	JAK2	−11.71	Decrease	−2.71316	8	0.0265
Hour 1	MAPK1	−51.04	Decrease	−3.09134	8	0.0149
Hour 1	MAPKAPK2	−33.95	Decrease	−2.48863	8	0.0376
Hour 1	MARCO	−15.47	Decrease	−2.62623	8	0.0304
Hour 1	NOD2	25.29	Decrease	2.38436	8	0.0442
Hour 1	NOTCH2	−95.69	Decrease	−3.02181	8	0.0165
Hour 1	PRKCD	−24.65	Decrease	−2.39875	8	0.0433
Hour 1	SLAMF7	−9.59	Decrease	−3.12773	8	0.0141
Hour 1	STAT3	−88.35	Decrease	−2.35686	8	0.0462
Hour 1	IL20	15.61	Increase	3.18543	8	0.0129
Hour 6	BCL3	67.53	Increase	3.040491	7	0.0188

*Nanostring quantified RNA transcripts were sampled at 1 and 6 h post-LTA stimulation*.

## Discussion

Our results show that *in vivo* exposure to CS alters the secretion of key antimicrobial peptides from subsequently cultured PHNEC. We observed a significant decrease in apical and basolateral SLPI, and BD-1 secretions as well as a reduction in apical secretions and an increase in basolateral secretions of CCL-20 from PHNEC cultured from CS-exposed individuals. As these are important antimicrobial peptides, it shows that CS may decrease the overall antimicrobial activity of nasal secretions. Previous work from our lab has shown that CCL20 constitutes an important fraction of antimicrobial activity in the secretions of airway (BEAS2B) cells (34). PHNEC from CS-exposed individuals were more difficult to culture than that from non-smoke-exposed individuals. Clearly, the cells that grow out to form PHNEC cultures have modifications that reflect CS exposure *in vivo*. These modifications could be epigenetic marks. This has implications for the time course of recovery of normal resistance to airway pathogens after smoking cessation.

The *in vitro* time-course data of LTA-stimulated PHNEC support other research showing that *in vitro* CS exposure alters the innate immune system or suppresses its ability to respond to pathogens. For example, Manzel et al. (37) showed that CSE suppressed the activation of NF-κB-dependent pathways, reduced the consequent expression of defense genes [IL-8 and intracellular adhesion molecule 1 (ICAM-1)], and decreased cellular secretion of IL-6 and IL-8 in the response to *Haemophilus influenzae* by primary human tracheobronchial epithelial cells. Also, cellular secretion of IL-8 and IL-6 were suppressed (37). Despite the fact that these same molecules did not have reduced expression in our transcription studies, these results in addition to ours suggest that exposure to CSE weakens the innate immune response of the upper airway mucosa to a bacterial invasion. The lack of full correlation between the studies could be due to different anatomical sources of primary epithelial cells, different stimulation conditions, or different culturing techniques.

Examining the list of genes that are significantly decreased in LTA-stimulated PHNEC with CSE exposure reveals that many are involved in immune signaling. Using the KEGG pathways, database shows that four of the genes occur in the neurotrophin signaling pathway (IRAK1, PKCD, MAPK1, and MAPKAPK2), four occur in the chemokine signaling pathway (JAK2, STAT3, MAPK1, and PKCD), two occur in the TLR signaling pathway (IRAK1 and MAPK1), and three occur in the JAK-STAT signaling pathway (IFNAR1, JAK2, and STAT3) (38). Macrophage Receptor (MARCO) is a class A scavenger receptor, probably involved in binding Gram-positive and Gram-negative bacteria (39, 40). SLAMF7 was also detected as a significantly down-regulated gene and SLAM receptors are involved in the fine-tuning of immune cell activation (41). NOD2 expression was increased and this gene is an intracellular receptor involved in the recognition of bacterial peptidoglycans and is involved in activation of NF-Kappa B, cytokine production, and apoptosis (42). Based on the gene expression data, CSE disrupts multiple chemokine, neurotrophin, and immune signaling pathways in PHNEC.

Our data suggest that individuals vary greatly in the amounts of antimicrobial peptides secreted by their PHNECs under baseline conditions. Constitutive and LTA-induced antimicrobial secretions vary greatly among both non-smoke-exposed individuals and *in vivo* CS smoke-exposed individuals. For example, PHNEC from non-smoke-exposed individuals varies approximately 60-fold from 19 to 1223 pg/ml in their secretion of CCL20 under control conditions and approximately 250-fold from 32 to 8000 pg/ml in LTA-stimulated conditions (data not shown).

We had increased difficulty in culturing PHNEC from CS-exposed individuals, suggesting that there are other phenotypic differences as well, perhaps at the level of the stem cells that grow out to form the PHNEC cultures. We could obtain nasal epithelial cells from these individuals, but they would stop dividing and would not proliferate enough to utilize experimentally. This along with our data, showing decreased secretion of antimicrobials from PHNEC from smoke-exposed individuals, suggests some transmissible epigenetic changes in CS-exposed epithelial stem cells from which the PHNEC are derived; these observations warrant further study.

The study was performed with PHNEC samples that had been cultured to a solid monolayer and then differentiated into mucociliary epithelial cells that, like *in vivo* airway epithelial cells, had distinct differences between the apical and basolateral surfaces. Variability in response to secretion of the assayed proteins/peptides between basolateral and apical surfaces was revealed in some conditions in which the same cultures responded differentially between apical and basolateral secretion (for example, see Figure 2). This suggests that posttranslational regulation is affected by CS exposure. Since basolateral or apical secretion has different implications for fighting infections, it is likely that the different secretion impacts also play a role in sensitivity of smoke-exposed individuals to airway infections.

Antimicrobial peptides and proteins are important in protecting humans from respiratory pathogens that smoke-exposed people are less able to control. Our results reveal clear suppressive effects of both *in vitro* and *in vivo* CS exposure on some of these defensive peptides and changes in the expression of genes involved in chemokine and neurotrophin signaling pathways. It is not clear why there are disparate effects on different specific proteins and peptides and an understanding of those issues requires further study. This report contributes to the mechanistic understanding of how CS exposure alters the innate immune response and increases an individual’s susceptibility to pathogen infection.

## Author Contributions

JJ was central to this project with involvement in all aspects of research, including cell culture and assays, data analysis and statistics as well as having primary responsibility for figures and manuscript. BG trained JJ and MC-G to harvest tissues from study volunteers and collaborated on research design. TD did cell cultures and assays. LF helped with assays and data analysis. SF and MC-G mentored research design and development of cell culture protocols and research skills as well as in data analysis and manuscript development.

## Conflict of Interest Statement

The authors declare that the research was conducted in the absence of any commercial or financial relationships that could be construed as a potential conflict of interest.
